# Breaking the Silence: Addressing Domestic Abuse in Mental Health Settings—Identification, Screening, and Responding

**DOI:** 10.1177/15248380241280092

**Published:** 2024-10-08

**Authors:** Ema Baukaite, Kate Walker, Emma Sleath

**Affiliations:** 1 Northamptonshire Healthcare NHS Foundation Trust, Northampton, UK; 2 University of Leicester, UK

**Keywords:** domestic abuse, intimate partner violence, screening, disclosure, mental health service

## Abstract

Individuals experiencing domestic abuse (DA) struggle to disclose victimization, but as frequent users of mental health services, this is a pivotal setting for identification and addressing DA. This systematic review of 20 studies investigates DA identification, screening, and responses within mental health settings. Three databases were searched using these inclusion criteria: adults aged 18 and older accessing mental health services, samples comprising mental health professionals (or combination). No geographical restrictions were applied. All studies were peer-reviewed and published in English between January 2000 and December 2023. Studies had to incorporate screening for DA between (ex-)partners and/or response to disclosure within mental health settings. The findings revealed considerable variation in DA screening methods from direct screening tools to retrospective analyses of patient files. Professionals report barriers in identifying DA, including uncertainty about their role, time constraints, and the importance of building trust with service users. Nonetheless, many highlight the importance of routinely asking about DA. A small number of interventions have been effective in enhancing professionals’ readiness to address DA, but it remains unclear what format of training is most effective. Service users report feelings of shame and fear of not being believed when disclosing DA, but are aided by therapeutic engagement and enhanced professional awareness. There is a lack of diverse inclusion in the research. In summary, there is considerable scope to develop good practice to support mental health professionals’ ability to identify and respond to DA across assessment tool and training, but also in understanding what facilitates service users to disclose.

## Introduction

Domestic abuse (DA) encompasses incidents of controlling, coercive, or threatening behavior, as well as violence or abuse among family members or intimate (ex-)partners aged 16 or over (National Institute for Health and Care Excellence [NICE], 2016). The nature of this abuse spans psychological, physical, sexual, financial, and emotional abuse, including “honor”-based violence and forced marriage (NICE, 2016). While DA is commonly used interchangeably with intimate partner violence (IPV), in this review, the term DA will be used and specifically refers to acts of abuse occurring within (ex-)intimate partnerships (Cunradi, 2010; Patra et al., 2018).

DA is widespread; over one in three women have experienced DA or non-partner sexual violence at least once in their lives (World Health Organization [WHO], 2024). While women are disproportionately affected (Hasstedt & Rowan, 2016), men also experience DA; in English-speaking nations, nearly one in five men have faced physical violence from an intimate partner (Breiding et al., 2010; Desmarais et al., 2012). As a pervasive societal issue, 6.9% of women and 3.0% of men reported DA in the past year in England and Wales (Office for National Statistics, 2022), but under-reporting is significant (Felson et al., 2002). Moreover, variations in DA across different racial/ethnic groups, individuals’ sexual orientation, and disabilities have been documented (Niolon et al., 2017), with higher lifetime DA prevalence among ethnic minority women, bisexual, lesbian, and gay individuals, and those with disabilities, particularly physical and mental health impairments (Niolon et al., 2017).

DA adversely affects physical health and has also been linked to a range of mental health impacts, including depression, anxiety, psychosis, schizophrenia, bipolar disorder, and serious mental illness (Chandan et al., 2020), post-traumatic stress disorder (Jones et al., 2001), and eating disorders (Bundock et al., 2013). Lifetime experience of any type of DA is associated with suicidal ideation, psychological distress, and depression in women (White et al., 2023). Both men and women experiencing physical DA face an increased risk of depressive symptoms, substance use, and the development of chronic mental illness (Coker et al., 2002). There exists a bidirectional relationship between DA and mental health, where mental health issues increase vulnerability to DA (Trevillion et al., 2012), and DA negatively impacts mental health (Devries et al., 2013; Howard et al., 2013; Loxton et al., 2017).

Research has focused on a range of healthcare settings (e.g., general practice, maternity services, sexual health, substance misuse, child and vulnerable adult services, and mental health services (NICE, 2016), where it is important to ask service users about their potential experiences of DA (also called screening for DA), to understand what are the best ways to implement this, and then how to respond when disclosures are made (e.g., IRIS project, Sohal et al., 2020). In understanding the different approaches that can be taken when asking about DA, WHO (2013) guidelines emphasize that “universal screening” or “routine enquiry” (i.e., asking *all* women in *all* healthcare encounters) should not be implemented. However, they propose that it is good clinical practice to specifically ask all women with mental health issues about DA, particularly as this may affect their treatment and care (WHO, 2013). Moreover, NICE recommends that professionals in mental health settings routinely inquire about DA, even in the absence of indicators of violence and abuse, because survivors of DA frequently use these services (Friedman & Loue, 2007; Heron & Eisma, 2021; Howard et al., 2009; Oram et al., 2013). As such, the Department of Health (2008) in the United Kingdom has implemented a protocol for regular examination of DA within mental health environments.

These policy and practice guidelines clearly advocate for the inclusion of DA screening as part of standard evaluations in mental health settings (see also Trevillion et al., 2016); however, research demonstrates that mental health professionals do not consistently inquire about DA, and service users often do not volunteer information about DA unless specifically asked, and even then, may choose to not disclose (e.g., Howard et al., 2009). This results in DA frequently being unnoticed and unaddressed within mental health services (e.g., Chang et al., 2011; Doyle et al., 2022; Howard et al., 2009).

The effectiveness of DA screening is evaluated through a number of outcomes including the identification of DA, increased referrals to support services, reduced re-exposure to violence, and improved health outcomes (O’Doherty et al., 2015). In understanding why effective DA screening may not be occurring, mental health professionals have reported barriers including an inadequate understanding of DA, feelings of personal inadequacy, unfamiliarity with available resources, and a deficit in training and knowledge related to effective clinical practices for managing DA (Dichter et al., 2015; Forsdike et al., 2018). There is also a lack of validated or standardized screening tools with a review by Arkins et al. (2016) identifying only three comprehensive DA screening tools validated against appropriate standards. As these tools were originally developed for cisgender women in heterosexual relationships, questions arise regarding their validity, reliability, and effectiveness for use in diverse populations, for example, transgender (Peitzmeier et al., 2020). Peitzmeier et al. (2020) also note that healthcare providers often lack training in interviewing this population, compromising the identification of experiences of DA. Notably, a recent review by Gómez-Fernández et al. (2019) listed tools for detecting DA in clinical settings, and while they highlighted their suitability in emergency services, they did not address their suitability for DA screening in mental health settings. Ultimately, this failure to identify and thus be able to respond to disclosures of DA in mental health settings indicates a poor understanding of its connection to mental illness.

### Research Gap and Aim of the Study

Previous reviews have examined how mental health services respond to DA, covering aspects such as identification, referral procedures, and the provision of care (e.g., Trevillion et al., 2016) and the experiences of service users disclosing DA (Trevillion et al., 2014). However, these past reviews focus predominantly on female service users and lean heavily toward qualitative research on victims’ disclosure experiences (Trevillion et al., 2014). No review has evaluated the effectiveness of DA screening tools in relation to how well they may work in real-world mental health settings (see Gómez-Fernández, 2019, who discusses this in relation to clinical environments, but not specifically mental health settings). This review aims to evaluate available screening methods that have been used in mental health settings in relation to their validity and reliability in identifying DA. Additionally, it will assess whether these screening methods increase referrals to support services, reduce re-exposure to violence, and improve health outcomes. Furthermore, no review has triangulated perspectives from both service users and mental health professionals, providing a more comprehensive and nuanced view of how issues are likely to intersect. Our systematic review will address these gaps, and in doing so, we will highlight findings from diverse samples of service users and/or mental health professionals, to elevate our understanding of key issues beyond female service users, but also to highlight gaps across other minority status groups in relation to DA within the context of screening in mental health settings. From these findings, we will develop practice-based recommendations to drive enhancements in this sector, ultimately to benefit survivors of DA. As such, this review will explore the tools that have been used in the literature in mental health environments for detecting, screening, and addressing DA, with a specific focus on their relevance in real-world practice settings. Building upon previous research, this review also outlines the measures of DA management and response, examining challenges and barriers, while systematically evaluating the quality of the studies. Specifically, it aims to address the following questions:

(a) What tools are currently employed in the research literature in mental health settings for identifying, screening, and responding to DA, and how appropriate are they for use in real-world practice settings?
(b) What are the differences in DA screening practices and outcomes among different demographic groups (e.g., men, women, LGBTQ individuals) within mental health settings?
(c) How do mental health professionals and service users perceive DA screening?

## Methods

This systematic review adhered to the Preferred Reporting Items for Systematic Reviews and Meta-Analyses (PRISMA) guidelines (Page et al., 2021).

### Eligibility Criteria

Both qualitative and quantitative studies were included in this review. Eligible study populations included: adults aged 18 and older accessing mental health services, samples comprising mental health professionals, or a combination of both. No geographical restrictions were applied. All studies were peer-reviewed and published in English language between January 2000 and December 2023. Inclusion criteria required studies to incorporate screening for DA between (ex-) partners and/or response to disclosure within mental health settings.

Excluded studies encompassed several categories: (a) non-empirical studies (e.g., letters to editors, discussion papers, commentaries, editorials, book reviews, book chapters, and general reviews), assessed using The Mixed Methods Appraisal Tool (MMAT) version 2018 (see Hong et al., 2018); (b) research screening for other forms of family violence, not focused on DA between (ex-)partners; (c) studies not focused in mental health settings, or lacking clear delineation of mental health settings or failing to explicitly distinguish mental health services; (d) studies involving military or veteran samples; and (e) studies including participants aged 17 or younger.

### Search Strategy

The selected studies were identified using three electronic databases, PubMed, MEDLINE Complete (EBSCO), and PsycINFO. A search strategy was devised based on four primary component terms: (a) DA; (b) mental health settings; and (c) screening; and/or (d) disclosure. The search terms and keywords encompassed various facets of DA, such as domestic violence, IPV, spouse abuse, battered women, or battered men. See Supplemental material Appendix I for the complete list of search terms and the search process.

To ensure precision, comprehensiveness, and consistency, the initial search utilized Medical Subject Headings (MeSH) to accurately capture relevant articles (DeMars & Perruso, 2022). Subsequently, free text words were employed to ensure a comprehensive search, capturing relevant content that might not be covered by the subject terms. The initial search was conducted on PubMed, involving both MeSH terms and free text word searches to identify synonyms and pertinent terms related to the three main components. This process was iterated, incorporating additional terms identified in the initial search (last searched performed December 31, 2023).

### Study Selection and Data Extraction

**Figure 1.**
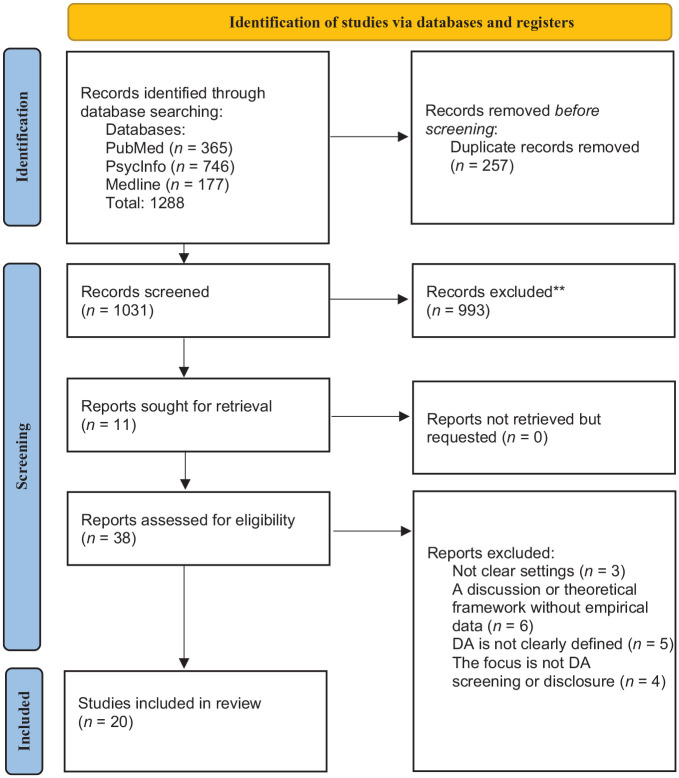
Summary of the identification and inclusion of relevant publications according to Preferred Reporting Items for Systematic Reviews and Meta-Analyses procedure.

See Figure 1 summarizing the identification and inclusion of relevant publications (PRISMA, 2009).

Screening for eligibility was carried out by evaluating the titles and abstracts against the criteria (by the first author). In instances where eligibility was unclear on this basis, the full text of the article was retrieved. Subsequently, for articles meeting the initial screening criteria, all three authors independently conducted a comprehensive review of the full texts to confirm eligibility. Any discrepancies were resolved through discussion and consensus. Relevant data from eligible articles were systematically extracted and documented in two dedicated spreadsheets. Tables were employed to display significant results in quantitative studies and themes, patterns and/or insights in qualitative studies organized via the following categories: screening for DA in mental health settings; variances or resemblances in DA screening practices across diverse populations; perceptions of DA screening by mental health professionals and service users; management of and response to DA; and minority status representation in samples of service users. Study quality was evaluated utilizing the MMAT (2018 version), a critical evaluation instrument tailored for systematic reviews incorporating qualitative, quantitative, and mixed methods (Hong et al., 2018). The first and second author performed this assessment to ensure a robust evaluation of study quality, with no discrepancies found between these analyses. The distribution of MMAT scores exhibited variability across different study designs, but the majority of the papers were categorized as high quality, as indicated by four “yes” ratings, with none categorized as low quality (one “yes”) (Pluye et al., 2009). On this basis, no papers were excluded from the review. Notably, all qualitative studies attained high-quality ratings, while only one quantitative study was deemed to be of average quality (Chandra et al., 2009).

## Results

The characteristics of the selected studies, including their types, settings (country and healthcare setting), focus areas, participant demographics and their mental health conditions, and summary of findings are presented in Supplemental material Appendix 2 for quantitative studies and Supplemental material Appendix 3 for qualitative studies. A table of the critical findings is presented in Table 1. For all three questions, we screened 1,031 studies from three databases: PubMed, PsycInfo, and MEDLINE. We attempted to retrieve 11 of these studies because they were not immediately accessible. Out of the 1,031 studies, we extracted the full text for 38 studies, including those we sought for retrieval. Ultimately, 20 studies were included in the final review: 12 from PubMed, 6 from PsycInfo, and 2 from MEDLINE.

**Table 1.** Critical Findings of the Review.

1. DA screening tools may be effective in identifying DA; however, questions remain regarding validation in diverse populations, limitations regarding assessing for different forms of DA, and finally how fit-for-purpose these tools are in practice settings.
2. Training professionals to identify and respond to DA can be effective in enhancing professionals’ perceptions of their ability to do this; however, it is unclear whether this improves the actual identification and response given to survivors.
3. Professionals identify training needs that necessitate more comprehensive approaches that extend beyond one-off training.
4. There is an extensive understanding of the numerous barriers that professionals and survivors experience in asking about, and disclosing DA. It is less clear what facilitates this process.

*Note*. DA = domestic abuse.

### DA Screening Tools in Mental Health Settings, and Their Validity and Reliability in Identifying DA

Six out of ten quantitative studies employed DA screening tools, covering at least three forms of abuse: physical, emotional/psychological, and sexual (Chandra et al., 2009; Chang et al., 2011; González Cases et al., 2014; Moodley et al., 2023; Trevillion et al., 2013; Vranda et al., 2020). None of these studies focused on evaluating the validity and reliability of the screening tools in identifying DA, however for four out of the six studies, there was evidence of good/acceptable reliability and validity in the measures used. For example, Moodley et al. (2023) highlighted for The Women Abuse Screening Tool (WAST), high internal consistency (Cronbach’s α: .75), sensitivity (92%), specificity (100%), and correlation with the Abuse Rating Inventory (*r* = .96) based on its original development (Brown et al., 2000). Vranda et al. (2020) assessed partner abuse using the IPV Screening Questionnaire and the Danger Assessment Questionnaire, validated through linguistic and content validation and pilot testing involving 30 women. Chandra et al. (2009) used the Index of Spouse Abuse (ISA) and the Sexual Experiences Scale (Hudson & McIntosh, 1981), both previously validated and translated into Kannada. Trevillion et al. (2013) used the Composite Abuse Scale (CAS), citing the validity and reliability from previous research (Hegarty et al., 1999, 2005). These studies reported strong internal consistency (reliability) for the CAS, with a Cronbach’s α value of at least .90. For the remaining two studies, reliability and validity of the measures were not always as clear. González Cases et al. (2014) utilized the IPV toward Women Questionnaire, validated for the Spanish population (Lasheras et al., 2008) and Conflict Tactic Scales (CTS) (Straus, 1979), but its validation and reliability were not stated. Chang et al. (2011) assessed IPV victimization using various adapted questions from the Abuse Assessment Screen, Women’s Experience of Battering Scale (Smith et al., 1995), and from prior research (Currier & Briere, 2000), without specifying validation and reliability. Additionally, only three screening tools—WAST, employed by Moodley et al. (2023); CAS, utilized by Trevillion et al. (2013); and the IPV Screening Questionnaire, employed by Vranda et al. (2020)—covered at least three types of DA (physical, emotional, and sexual). Other studies required the use of multiple screening tools to assess different forms of DA.

### Other DA Identification Approaches Utilized in Mental Health Settings

In relation to less direct screening measures of DA, four studies used this approach (Burns et al., 2022a, 2022b; Ruijne et al., 2021, Spangaro et al., 2010), with varying levels of information provided regarding reliability and validity. Burns et al. (2022a, 2022b) employed online surveys to gage the perceived prevalence of physical or sexual IPV in clinical settings, where professionals rated their frequency of using Relationship Problem and Maltreatment codes (Burns et al., 2022a); and an online survey to detect mild forms of physical and psychological abuse in vignettes (Burns et al., 2022b). The Relationship Problem and Maltreatment codes underwent extensive field testing with a global sample of clinicians to ascertain their reliability and clinical utility (Burns et al., 2022a).

Ruijne et al. (2021) conducted a retrospective analysis without specifying the exact DA (sexual, physical, material, or emotional) screening method used by professionals. Spangaro et al. (2010) used scripted questions focused on experiences of mainly physical IPV. The study tested the survey and consulted with women who had experienced IPV to ensure the survey questions were understandable and minimally intrusive, but validation and reliability were not specifically noted or highlighted.

### Summary of DA Screening Methods and Their Effectiveness

Given the limited nature of the literature and, therefore, the small number of scales being used, this synthesis did not provide strong evidence of a stand-out tool that could be recommended for use in mental health settings when working with service users. The WAST and CAS emerged as the most validated screening methods, showing high internal consistency and sensitivity. However, the CAS is not a screening or clinical tool but was developed as a research tool to measure all types of partner abuse (Hegarty et al., 2005). Although the CTS is a widely used tool and is reported as a reliable and valid measure of DA across different populations and cultures, Chapman and Gillespie (2019) suggest that more research is required regarding its validity and reliability in clinical and forensic settings; this therefore needs to be examined and reported before recommending its use in mental health settings. Other methods like the IPV Screening Questionnaire and the ISA showed promise but lack comprehensive validation. Chang et al.’s study highlighted significant gender differences in IPV victimization, suggesting potential applicability of the tool for both genders. However, scripted questions, retrospective analysis, and online surveys with established guidelines did not efficiently cover all forms of DA and lacked sufficient validity and reliability.

### Sample Characteristics Across Studies

Across the 11 studies involving service users, 967 females and 167 males participated. Only four studies included both genders (Chang et al., 2011; Rose et al., 2011; Trevillion et al., 2012, 2013). However, three of these studies had significantly fewer male participants compared to females (Rose et al., 2011; Trevillion et al., 2012, 2013). Only the study by Chang et al. (2011) included 158 males and examined screening practices for both genders, revealing that women were twice as likely as men to recall being asked about all forms of DA. The remaining seven studies focused exclusively on female participants.

In relation to sexuality, Trevillion et al. (2013) defined DA as occurring between partners or family members irrespective of their gender or sexuality but did not report findings relating to differences in screening practices among service users of different sexualities. The remaining studies either specified that they were examining DA experienced in heterosexual relationships or did not provide information on the sexuality or type of relationships of their service users’ sample.

Six studies did not provide any ethnicity data and three studies had incomplete data (Chang et al., 2011; Moodley et al., 2023; Rose et al., 2011), meaning that ethnicity information was not reported for 687 service users. For those studies that did report ethnicity, service users were predominantly of White/European descent or Caucasian/White (*N* = 284), followed by Black, Black/African descent, or Black/Caribbean descent (*N* = 87). Other reported ethnicities included Hispanic/Latino (*N* = 2), Indian (*N* = 32), Colored/Mixed Race (*N* = 19), Asian (*N* = 2), and one study combined black and ethnic minority (*N* = 20). However, none of these studies examined differences in screening practices for different ethnic groups.

Studies included individuals presenting with a wide range of mental health conditions, encompassing both severe (Moodley et al., 2023) and minor psychiatric disorders (Chandra et al., 2009). Different mental health presentations included: affective and non-affective psychiatric disorders (Vranda et al., 2020); schizophrenia, personality disorder, bipolar disorder, and other psychotic disorders (González Cases et al., 2014; Trevillion et al., 2013); borderline personality disorder and adjustment disorder (Rose et al., 2011; Trevillion et al., 2012); somatoform disorder, depression, anxiety, and minor psychiatric disorders (Chandra et al., 2009; Chang et al., 2011; Rose et al., 2011; Trevillion et al., 2013); and some service users currently in remission (Vranda et al., 2018). These studies collectively identify a lack of diversity in the research in relation to demographic characteristics, but have involved a broad spectrum of mental health conditions prevalent in those with experience of DA. There was no differential screening for DA practice applied across the different mental health presentations.

### Evaluating Training for Professionals on Screening and Response Practice

Four studies examined intervention or training programs for mental health professionals to improve their DA response and/or screening skills (Lloyd et al., 2017; Ruijne et al., 2020, 2021; Trevillion et al., 2013). Two studies assessed the adherence of mental health professionals’ training to WHO guidelines and its impact on DA screening effectiveness (Burns et al., 2022a, 2022b).

Trevillion et al. (2013) used mixed methods to evaluate a multifaceted approach to address DA within community mental health teams (CMHT). Professionals received 4 hrs of specialized Linking Abuse and Recovery through Advocacy (LARA) intervention, covering domestic violence identification and response, supported by a comprehensive manual. Domestic violence advisors underwent 6 hrs of mental illness awareness training. A direct referral pathway to domestic violence advocacy services was established for timely support. Trained advisors provided integrated advocacy services, including emotional support and practical assistance. An information campaign raised awareness of DA and available support services among CMHT service users. The study employed validated measures and scales to assess various outcomes, including the Physician Readiness to Measure Intimate Partner Violence Survey (PREMIS) for professionals and the CAS for service users (Short et al., 2006). Quantitative data from the intervention showed significant improvements including perceived preparation, knowledge, and self-efficacy for professionals. Service users in the intervention group reported experiencing significantly decreased violence. Qualitative data revealed that service users credited domestic violence advisors for reducing harm, received assistance for various needs, emphasized feelings of social inclusion through community engagement, and valued practical and emotional support from advisors.

Ruijne et al. (2021) employed a cluster randomized trial and developed an intervention The Better Reduction and Assessment of Violence (BRAVE). Based on the LARA intervention, but adapted to the Dutch context (Ruijne et al., 2017; Trevillion et al., 2013), its goal was to enhance the detection, referral, and support for both male and female victims of DA, while also considering potential cultural differences. Additionally, a fidelity assessment conducted by an independent researcher evaluated the presence of core intervention components over the intervention period. While the fidelity scores ranged from 6 to 11, indicating varying levels of adherence, this assessment provided insights into the reliability of intervention delivery across teams. As found in Trevillion et al. (2013), significant improvements in professionals’ readiness to manage DA were observed after undertaking the BRAVE intervention; however, it did not result in an increase in detection and referral rates. This latter finding may reflect that detection and referral rates were assessed through a retrospective analysis of clinical files, which may not have captured all the data accurately. Professionals reported positive feedback of the BRAVE intervention, finding it feasible and that it enabled them to use the learned skills. However, there remained a lack of confidence among professionals in applying their knowledge in practice. Professionals also reported a desire for peer consultation or a dedicated DA consultant for emotional support. As such, the authors concluded that sustaining effective implementation of DA knowledge and skills requires more than “one-off” training. These findings mirror those reported by Ruijne et al. (2020) in their pilot examination of professionals’ views of the BRAVE intervention.

Lloyd et al. (2017) evaluated an intervention program that aimed to train mental health providers to better identify, manage, and respond to cases of DA. The training program comprised four to eight 1-day sessions, focusing on inquiry techniques via roleplay and intervention models for families, couples, and individuals, including referral processes. Program effectiveness was assessed through quantitative and qualitative methods at pre-program, immediate post-program, and 6-month follow-up stages, with mental health professionals. The findings, via using the PREMIS survey, conducted 6 months post-program, showed mental health professionals had significantly improved in their perceived ability to identify victims of IPV and their readiness to discuss abuse with victims. This parallels the findings of both Trevillion et al. (2013) and Ruijne et al. (2021) in demonstrating that interventions can strengthen the perceived ability to identify DA and the readiness to respond by mental health professionals. Qualitative data identified key elements of the training program to ensure effectiveness including: a safe training environment for open discussion, the value of practical examples and first-hand survivor accounts, and the need for longer and more comprehensive training sessions. This latter point aligns particularly with the findings of Ruijne et al. (2021) in relation to one-off training. Participants also stressed the significance of multi-agency collaboration to address DA effectively, expressing concerns about resistance within professional circles and a lack of interagency cooperation, including with the police.

Burns et al. (2022a) assessed the extent to which mental health professionals’ training follows WHO recommendations and how it affects the professionals’ knowledge about relationship problems experienced by couples. A follow-up study by Burns et al. (2022b) examined how it improves the accuracy of identifying these relationship problems. An online survey was used to assess perceived frequency of DA in clinical practice, DA related training, and knowledge of relationship problems (2022a); a second online survey assessed usage of ICD guidelines and vignettes to evaluate and assess professionals’ ability to diagnose relationship problems (2022b). These two studies found no difference in DA identification accuracy between users of ICD-11 and ICD-10 guidelines (2022b), and that years of professional experience, DA-related laws, perceived frequency of DA in clinical practice, and experiences of DA-related training, all predicted professionals’ DA knowledge (2022a). Professionals with DA-related training were significantly more able to correctly respond when identifying relationship and maltreatment problems compared to those without training, emphasizing the importance of professionals having relevant training. Training hours did not significantly predict the likelihood of higher numbers of correct responses, somewhat countering the views by professionals in Lloyd et al. (2017) that longer training would be more beneficial. However, this result was undermined by the finding that professionals who received three or four components of training performed significantly better than those without training, but those receiving only one or two components of training did not demonstrate improved performance compared to no training, emphasizing the point that *comprehensive* training is likely to be more effective.

In summary, these studies demonstrate that training can support professionals to more effectively identify DA; however, the limited nature of these studies means that identifying the key components of training is currently unclear. Furthermore, the impact of training professionals on the effectiveness of DA identification and the pathway to subsequent reductions in DA experienced by service users has not been established. The training programs were broadly similar (particularly since BRAVE is based on LARA) in focusing on developing skills regarding talking to and direct communication with patients and enhancing knowledge regarding DA (Ruijne et al., 2020, 2021; Trevillion et al., 2013). Lloyd et al. (2017) also used this approach, but was slightly more directive in providing specific examples of inquiries about DA that professionals could use with clients, embedded through the use of roleplay.

### Mental Health Professionals’ Views on DA Screening and Responding

Seven qualitative studies (Donnelly & Holt, 2020; Gillespie et al., 2022; Rose et al., 2011; Ruijne et al., 2020; Spangaro et al., 2011; Trevillion et al., 2012; Wilson et al., 2021) and one quantitative study (Nyame et al., 2013) captured mental health professionals’ views on DA screening and responding, which consisted of barriers and facilitators. Nyame et al. (2013) utilized the PREMIS survey to examine mental health professionals’ ability to identify and respond to DA. While female professionals demonstrated greater initiative in assessment and management of domestic violence, no other significant gender variances were observed in other PREMIS sub-scales. Psychiatrists showed significantly better knowledge in relation to DA, while nurses were significantly more ready to respond and take action in relation to DA.

The qualitative studies reported a range of barriers experienced by professionals, in relation to identifying DA including: privacy concerns and professionals’ frustration with women who choose to stay in abusive relationships (Spangaro et al., 2011); limited access to training, lack of prioritization of DA screening, and mental health overshadowing DA inquiries (Gillespie et al., 2022); role uncertainty and time constraints, fear of causing offense, and uncertainty about which questions to pose or how to manage the information (Rose et al., 2011; Trevillion et al., 2012); practical challenges such as intake protocols lacking inquiries about DA; emotional barriers such as hesitation to address DA prematurely or in the presence of the alleged perpetrator, and patient disengagement or negative reaction (Ruijne et al., 2020); and concerns about appropriateness and sensitivity of routine screening during acute illness episodes, service users not recognizing the abuse, professionals’ lack of understanding, and dominance of a biomedical model (Donnelly & Holt, 2020). Wilson et al. (2021) categorized barriers into individual, relational, community, and societal, including personal struggles, reluctance to disclose, and systemic issues hindering effective counseling. Across the studies, one of the most reported barriers to DA screening among mental health professionals was a lack of training and resources.

Across the studies reviewed, only one study (Spangaro et al., 2011) specified the DA screening method used by professionals, in this case the New South Wales Health Screening scripted questions. Without knowing the specific screening approach used by mental health professionals in the studies, it can be challenging to definitively identify the most significant barrier. As the effectiveness of different screening approaches can vary significantly, barriers reflect the specific methods being employed. Thus, comprehending the screening approaches employed could illuminate whether different barriers are linked to varying screening methods.

Only two studies identified facilitators to asking about DA and professionals reported these as: routine enquiry with efficient questions, structured forms, and visually presenting the questions to the patients (Spangaro et al., 2011), therapeutic engagement (Rose et al., 2011), and routine inquiry incorporated into clinical assessments (Trevillion et al., 2012).

Donnelly and Holt (2020) explored barriers to responding to DA and found that professionals perceived DA as falling beyond the scope of mental healthcare and considered it primarily a domain for social work. Trevillion et al. (2012) found that integrating discussions on DA into routine practice and being sensitive and available to service users when identifying DA were reported as facilitators to responding to DA by professionals. While some professionals encountered challenges in accessing referral services, others experienced positive interactions marked by effective communication and support from specialized agencies.

### Mental Health Service Users’ Views on DA Disclosure

Three qualitative and two quantitative studies focused on mental health service users’ perspectives on DA disclosure, examining facilitators and barriers, and expectations. These studies revealed various barriers to disclosing DA among mental health service users, including shame, fear of not being believed, threats from partners and family, fear of re-traumatization, and skepticism (Rose et al., 2011; Vranda et al., 2018). Spangaro et al. (2010) explored barriers with a sample of female participants, who had experienced recent or current abuse, but chose not to disclose even when specifically asked. Reasons for not disclosing included underestimating the seriousness of the abuse, fear of the offender discovering the disclosure, discomfort with healthcare providers, embarrassment, shame, and concerns about others finding out. However, there is a lack of perspectives from male participants, which complicates the understanding of gender-specific barriers to disclosure. For example, Rose et al. (2011) did not differentiate their findings regarding the barriers (or facilitators) to DA disclosure by gender.

Only two studies explored facilitators of DA disclosure, highlighting the importance of therapeutic engagement between professionals and service users and routine inquiry incorporated into clinical assessments (Rose et al., 2011; Trevillion et al., 2013).

Vranda et al. (2020) examined service users’ expectations from mental health professionals but did not provide detailed information on the measure construction. They identified primary needs among women affected by violence, including counseling services for psychological distress, stress management support, police assistance, treatment for abusive partners’ alcohol problems, access to shelter care facilities, family counseling, and anger management skills for abusive partners. Chandra et al. (2009) included a measure to ask about patients’ perceived reasons for their abuse; however, the results were not reported. This lack of clarity and reporting impedes our understanding of service users’ specific needs and expectations; this is crucial for informing interventions and support.

## Discussion

As a critically important setting for the identification of DA, this novel review focused on mental health settings, and summarizes the current literature regarding DA identification methods, mental health professionals’ perspectives on DA screening and response, mental health service users’ views on DA disclosure, and interventions for professionals aimed at improving DA screening and response. In all, this revealed a limited body of literature and where there is considerable scope to develop our understanding of good practice in relation to identification and responses to DA. Our review emphasizes the need for action over merely reporting results, highlighting significant gaps in the current literature. As shown in Table 2, while existing studies provide valuable insights, they often fall short in addressing the specific needs of diverse populations and the practical implementation of screening tools. This underscores the necessity for more comprehensive research that not only examines outcomes but also identifies effective practices, training, and interventions tailored to the unique challenges faced by mental health professionals and service users alike. By focusing on these needs, we aim to pave the way for future studies that will better inform policy and practice in the field of DA identification and response within mental health settings.

**Table 2.** Implications for Practice, Policy, and Research.

Practice
● Professionals need a holistic *identification and response framework* that they can embed in their practice and effectively support survivors of DA. This is anticipated to need to comprise the following components: ● Given the high likelihood of DA survivors accessing mental health services, organizations need to have effective screening and identification methods and processes that enable survivors to disclose. ● Professionals need access to comprehensive training that overcomes the barriers of asking about DA and enhances their readiness to address DA and be able to respond when service users disclose. ● Culturally sensitive approaches to DA screening and intervention need developing and implementing in practice to address diverse needs and backgrounds of service users.
Policy
● Engagement with DA survivors as those with lived experience to ensure that any DA screening and response framework is survivor-focused and reflects the diverse needs and nature of DA survivors. ● Encourage the inclusion of DA screening protocols and audits to ensure that these are embedded effectively into practice.
Research
Avenues for future research include: ● Examining the tools or screening methods that mental health professionals employ to identify DA; examine the suitability of available screening options in their settings; explore the rationale behind their choices; and assess the appropriateness of these methods in mental health settings. This would offer valuable insights into professionals’ decision-making processes, the suitability of screening methods that could contribute to the refinement and in turn efficacy of screening practices in mental health settings. ● The identification or creation of dependable screening tools that accommodate the diverse populations encountered in this context, thereby equipping mental health services with effective and validated tools to identify all potential survivors of DA. ● Longitudinal studies to assess the effectiveness of screening protocols and interventions for improving service users’ outcomes and to reduce the prevalence of DA. This research should ensure that any screening and intervention approaches are inclusive and culturally sensitive representing populations who are at higher risk of experiencing DA. ● Provide a thorough examination and identification of the barriers and facilitators to DA disclosure for service users, and asking about DA for mental health professionals ● Future studies should ensure balanced gender representation, explore diverse sexual orientations and relationship types, collect comprehensive ethnicity data, and utilize intersectional approaches to better understand DA screening practices and support diverse populations.

*Note*. DA = domestic abuse.

Selected quantitative studies included DA screening tools and alternative approaches (e.g., retrospective analysis and perceived frequency of DA detection) to examine DA occurrence in mental health settings. Notably, except for one study, qualitative research did not indicate or explore the screening approaches used by mental health professionals when examining their perception of screening. Therefore, in the context of DA within mental health settings, “screening” might be a misleading term. Traditional DA screening typically involves the use of standardized tools or structured questions aimed at identifying victims of abuse. However, the term might not fully capture the complexity and variability of methods used by mental health professionals. It could be that the term “identification” better captures the approach that professionals use with service users in exploring experiences of DA. While we know that DA screening/identification is taking place within some mental health settings, what constitutes good practice is limited by the absence of studies considering the specific methods (such as screening tools, standardized questions, or general unscripted questioning) used by mental health professionals to identify the occurrence of DA. This hinders our ability to determine whether these tools and approaches are routinely employed or if alternative identification methods are preferred (Nelson et al., 2012). Furthermore, out of six studies utilizing DA screening tools, the WAST demonstrated the highest validity, indicating potential suitability for implementation in direct screening initiatives in practice. However, it remains uncertain to what degree these tools would be feasible and applicable in real-world mental health settings, particularly given their predominant usage in cisgender female populations (Arkins et al., 2016). An additional issue is that the majority of screening tools do not assess the multiple ways in which DA can be enacted and instead predominantly focus one form of violence (primarily physical). Consequently, most studies employing screening tools had to utilize more than one tool to assess multiple forms of violence. Only three tools, included in three studies (Moodley et al., 2023; Trevillion et al., 2013; Vranda et al., 2020), covered multiple forms: emotional, physical, and sexual, leaving gaps in our understanding of the assessment of other forms of DA such as controlling behaviors and financial DA. Using additional tools increases the challenges to the feasibility of using these tools within practice, again raising issues that need to be further addressed. Then, finally, as indicated in the recent review by Velonis et al. (2021), the majority of screening tools continue to primarily focus on women and there is a lack of validated screening tools for men, as revealed by this review. In all, this review raises substantial questions about the utility of these screening tools and their application with service users in mental health settings.

In terms of addressing the second research question regarding differences in DA screening practices and outcomes among different demographic groups (e.g., men, women, LGBTQ individuals) within mental health settings, this cannot be fully answered at this point with the limited literature. Nearly all the studies involved samples of service users exclusively featuring females, meaning that there is a notable gap in research about DA among males more generally but specifically, a dearth in relation to mental health settings (Wörmann et al., 2021). The studies also predominantly focused on abuse within heterosexual relationships, with limited data on same-sex relationships. Only one study defined DA as occurring between partners regardless of their sexuality or gender; however, it did not specify whether their sample included any non-heterosexual participants. None of the other studies indicated that any of their samples included non-heterosexual individuals or those from LGBTQ+ or non-gender-conforming communities. Previous research indicates that sexual and gender minorities experience higher rates of DA and potentially more severe outcomes, leading to different trajectories (Beymer et al., 2017; Graham et al., 2016; McCauley et al., 2015). Researchers attribute this variability to minority stress, including sexuality-based discrimination and internalized homophobia, which affect DA experiences among LGBTQ+ individuals and heterosexual cisgender individuals differently (Stephenson & Finneran, 2016). There is a lack of studies that include LGBTQ+ samples or demonstrate their inclusion, indicating an insufficient focus on samples other than females (Decker et al., 2018); this therefore needs to be examined further. Alongside this, the limited ethnic representation within service users’ samples raises questions about cultural sensitivity and appropriateness of screening practices and training for professionals. Many studies did not report the ethnicity of the service users, which leads to the overrepresentation of individuals of White/European descent and highlights potential gaps in understanding and addressing DA among diverse ethnic groups. Racial discrimination correlates with higher mental health symptoms, which in turn relates to DA, particularly affecting Black women and those with lower incomes, indicating a necessity for interventions addressing the impact of discrimination on mental health and DA within the Black community (Maldonado et al., 2022). Also, sexual and gender minority people of color face higher rates of DA compared to white individuals (Whitton et al., 2021). Given the intersectionality of gender and ethnicity in shaping DA experiences, future research should prioritize inclusivity and cultural competence to ensure that screening and intervention efforts are tailored to the needs of diverse populations.

Furthermore, while the studies predominantly focused on service users with an active mental illness, not all patients were necessarily in this category. It is currently unclear whether DA identification practice should adapt according to the nature and complexity of mental health issues (e.g., co-morbidity), but future research should consider those with different mental health presentations to better understand this component of DA identification practice.

In terms of answering the third research question regarding how mental health professionals and service users perceive DA screening, the research coalesced around understanding barriers and facilitators to identification and responding to DA. Reported barriers to DA screening by professionals were numerous and this highlights the challenges to implementing effective practice in this area. Many of these link to a lack of comprehensive training for professionals in terms of understanding what good practice is in asking about experiences of DA, but also a lack of knowledge/skills about how to respond when disclosures occur. These triangulated with concerns from service users about how their disclosures might be responded to, and emphasized fear about re-traumatization, professionals’ failure to respond adequately, and discomfort with their clinician (Rose et al., 2011; Spangaro et al., 2010). Alongside this, mental health service users are not unique in also reporting embarrassment/shame, fear of being disbelieved, fear of being blamed, self-blame, all of which have long been recognized barriers to disclosing experiences of DA (Sylaska & Edwards, 2014). Understanding what did support identification and response by professionals and disclosure by services users was more challenging to identify, because of the limited nature of the literature; however, these did broadly center around the integration of asking about DA as part of routine/standard practice, advocated for by both professionals and service users, through direct questioning and engagement with professionals. However, studies generally lacked clarity on how service users’ expectations were measured, meaning that it is difficult to establish what are the key and important facilitators and barriers.

Given the concerns reported by professionals regarding the lack of training, the findings of this review based on the evaluation of three training programs for mental health professionals (Lloyd et al., 2017; Ruijne et al., 2020, 2021; Trevillion et al., 2013) are important. Somewhat similar in their approach to implementing the training, these focused on skill development and provided resources and practical tools for mental health professionals but differed in relation to duration, content focus, target audience, and delivery methods. The LARA intervention benefited from offering combined domestic violence and mental illness training for professionals and advisors, with direct referral pathways and an information campaign. The BRAVE intervention also included training for CMHT as well as domestic violence and abuse professionals, along with a direct care referral pathway, emphasizing collaboration. In all, these slightly different mechanisms regarding training need to be better understood to establish the best design and favored implementation to maximize effectiveness and ensure inclusivity. Overall, all three interventions showed some positive outcomes particularly in relation to professionals’ perceived ability in asking about and responding to DA; however, only the LARA intervention (Trevillion et al., 2013) was able to demonstrate professionals were using this training effectively, with service users in the intervention crediting the professionals for them having experienced significantly decreased violence. The effectiveness of training long term is also unknown as very little longitudinal research has been carried out. Finally, but of significant importance, is the lack of research demonstrating that these interventions are appropriate for diverse communities. While Lloyd et al. (2017) focused solely on DA identification and response in female service users, the LARA and BRAVE interventions were designed for both male and female service users. Beyond this, there is lack of knowledge regarding other diverse characteristics that service users represent and how this might affect the effectiveness of the training.

### Limitations

Due to time limits, only three databases were explored, which may limit scope of the studies included in this review. This narrow search strategy may not capture the full breadth of available literature on the topic. However, we ensured the comprehensiveness of the search strategy by including studies from diverse geographical locations, incorporating varied study types and methodologies, and including relevant participant groups, while also rigorously excluding studies that did not align with empirical research criteria on the topic.

In conclusion, this review emphasizes the importance of integrating DA screening and intervention into routine mental healthcare and the need for targeted efforts to address barriers to disclosure and facilitate access to support services. The implications for practice, policy, and future research are explored in Table 1 and represent important developments needed in this area, ultimately to better support survivors of DA.

## Supplemental Material

sj-docx-1-tva-10.1177_15248380241280092 – Supplemental material for Breaking the Silence: Addressing Domestic Abuse in Mental Health Settings—Identification, Screening, and Responding Supplemental material, sj-docx-1-tva-10.1177_15248380241280092 for Breaking the Silence: Addressing Domestic Abuse in Mental Health Settings—Identification, Screening, and Responding by Ema Baukaite, Kate Walker and Emma Sleath in Trauma, Violence, & Abuse
