# Supplementary material for: Breaking the Silence: Addressing Domestic Abuse in Mental Health Settings—Identification, Screening, and Responding
Source: Trauma Violence Abuse. 2024 Oct 8;26(3):436–50. doi: 10.1177/15248380241280092 (PMC12145471; doi:10.1177/15248380241280092)
Supplement: sj-docx-1-tva-10.1177_15248380241280092 – Supplemental material for Breaking the Silence: Addressing Domestic Abuse in Mental Health Settings—Identification, Screening, and Responding [file sj-docx-1-tva-10.1177_15248380241280092.docx]

**Appendix I**

For all searches, the primary keywords used were 'domestic abuse', 'mental health settings', 'screening', and 'disclosure'. Additional terms and MeSH term searches were built upon these keywords. Additional filters were applied as appropriate, as outlined in the accompanying table.

Table 1

*Literature Search Methodology Table*

| Database | Stage | Search type | | | MeSH Terms or Free text search | Results | Comments |
| --- | --- | --- | --- | --- | --- | --- | --- |
| Pubmed | 1. | MeSH | | | "Disclosure"[Mesh] OR "Mass Screening/psychology"[Mesh:NoExp] AND "Domestic violence"[Mesh] OR "Intimate partner Violence"[Mesh] AND "Mental Health Services"[Mesh] | 28 studies | Search found one relevant study, need to widen the search |
|  | 2. | Free text | | | ("domestic violence"[All Fields] OR "intimate partner abuse"[All Fields] OR "domestic abuse"[All Fields] OR "spouse abuse"[All Fields] OR "spouse violence"[All Fields]) AND ("mental health service*"[All Fields] OR "mental health setting*"[All Fields]) AND ("disclosure"[All Fields] OR "screening"[All Fields] OR "respon*"[All Fields]) | 128 studies, 5 repeated from 1st Stage, thus 122 results in total | Search found 18 potentially relevant studies |
|  | Results: The additional MeSH terms and keywords found during stages 1 & 2:  MeSH terms: "Clinical Competence/standards," "Attitude of Health Personnel," and "Professional-Patient Relations*,” "Clinical Competence/standards," "Attitude of Health Personnel," "Physician's Role," "Referral and Consultation," "Interview, Psychological," "Professional Role," and "Knowledge of Results, Psychological."  Keywords: "spousal abuse or violence" "intervention" "treatment" and "knowledge".  Additional terms 'detection' and ‘assessment’ were identified, which were synonymous with 'screening' in some titles of potentially relevant studies. | | | | | | |
|  | 3. | MeSH | "Disclosure"[Mesh] OR "Mass Screening/psychology"[Mesh] OR "Clinical Competence/standards"[Mesh] OR "Attitude of Health Personnel"[Mesh] OR "Physician's Role"[Mesh] OR "Referral and Consultation"[Mesh] OR "Interview, Psychological"[Mesh] OR "Professional Role"[Mesh] OR "Attitude of Health Personnel"[Mesh] OR "Knowledge of Results, Psychological"[Mesh] AND "Domestic violence"[Mesh] OR "Intimate partner Violence"[Mesh] AND "Mental Health Services"[Mesh] | | | 170 studies, 38 repeated, thus 132 in total | No relevant studies found |
|  | 4. | Free text | ("domestic violence"[All Fields] OR "intimate partner abuse"[All Fields] OR "domestic abuse"[All Fields] OR "spous* abuse"[All Fields] OR "spous* violence"[All Fields]) AND ("mental health service*"[All Fields] OR "mental health setting*"[All Fields]) AND ("disclosure"[All Fields] OR "screening"[All Fields] OR "respon*"[All Fields] OR "interven*"[All Fields] OR "treat*"[All Fields] Or "knowledge"[All Fields]) | | | 12 studies, 8 repeated, thus 4 in total | No relevant studies found |
|  | 5.  Automated filters: article type:   - Clinical Trial - Randomized - Controlled Trial - Clinical Study - Comparative study - Controlled clinical trial | Free text | ("domestic violence"[All Fields] OR "intimate partner abuse"[All Fields] OR "domestic abuse"[All Fields] OR "spous* abuse"[All Fields] OR "spous* violence"[All Fields] OR "battered women"[All Fields] OR "battered men"[All Fields] OR "intimate partner violence"[All Fields]) AND ("mental health service*"[All Fields] OR "mental health setting*"[All Fields]) AND ("disclos*"[All Fields] OR "screen*"[All Fields] OR "respon*"[All Fields] OR "detect*"[all fields]) | | | 9 studies, 2 repeated, thus 7 in total | 1 relevant study found |
|  | 6.  Automated filters: article type:   - Clinical Trial - Randomized - Controlled Trial - Clinical Study - Comparative study - Controlled clinical trial | Free text | ("domestic violence"[All Fields] OR "intimate partner abuse"[All Fields] OR "domestic abuse"[All Fields] OR "spous* abuse"[All Fields] OR "spous* violence"[All Fields] OR "battered women"[All Fields] OR "battered men"[All Fields] OR "intimate partner violence"[All Fields]) AND ("mental health service*"[All Fields] OR "mental health setting*"[All Fields]) AND ("disclos*"[All Fields] OR "screen*"[All Fields] OR "respon*"[All Fields] OR "detect*"[all fields] OR "assess*"[all fields]) | | | 18 studies, 9 repeated, thus 9 in total | No relevant studies found |
|  | Results: Initially, 365 studies were found, but after removing duplicates, the count reduced to 302. | | | | | | |
| Database | Search type | Key term | | MeSH Terms or Free text search | |  | |
| PsycInfo | MeSH | Domestic abuse | | "Marital Conflict"OR "Battered Females" OR "Intimate Partner Violence" OR "Domestic Violence" OR "Gender Violence" | |  |  |
| PsycInfo | MeSH | Mental health settings | | "Community Psychiatry" OR "Community Psychology" OR "Mental Health Services" OR "Community Mental Health Centers" OR "Community Mental Health" OR "Counseling" OR "Community Mental Health Services" | |  |  |
| PsycInfo | MeSH | Screening | | "Diagnosis" OR "Traumatic Experiences" OR "Psychiatric Evaluation" OR "Mental Health Screening" OR "Screening" OR "Trauma Screening" OR "Screening Tests" | |  |  |
|  | Results: Combined MeSH terms resulted in 37 studies. | | | | | | |
| PsycInfo | Free Text | Domestic Abuse | | domestic abuse OR domestic violence OR intimate partner violence OR intimate partner abuse OR battered women OR battered men OR spous* abuse OR spous* violence | |  | |
| PsycInfo | Free text | Mental health settings | | Mental health setting* or mental health service* | |  |  |
| PsycInfo | Free text | Screening or Disclosure | | disclos* OR screen* OR respon* OR interven* OR treat* OR knowledge | |  |  |
|  | Results: Combined free text terms resuled in 709 studies. Then, combined MeSH and free text terms resulted in 746 studies and 6 potentially relevant papers were identified. | | | | | | |
| Medline | MeSH terms | Domestic abuse | | (MH "Domestic Violence") OR (MH "Spouse Abuse") OR (MH "Gender-Based Violence") OR (MH "Intimate Partner Violence") OR "Domestic Abuse" OR (MH "Battered Women") | |  |  |
| Medline | MeSH terms | Mental health setting | | (MH "Mental Health Services") OR (MH "Counseling") OR (MH "Emergency Services, Psychiatric") OR (MH "Psychiatric Somatic Therapies") OR (MH "Psychotherapy") OR "mental health settings" OR (MH "Community Mental Health Services") OR (MH "Community Mental Health Centers") OR (MH "Mental Healing") OR (MH "Complementary Therapies") OR (MH "Psychiatric Rehabilitation") | |  |  |
| Medline | MeSH terms | Screening or disclosure | | "screening" OR (MH "Diagnosis") OR (MH "Diagnostic Techniques and Procedures") OR (MH "Diagnostic Screening Programs") OR (MH "Diagnostic Services") OR (MH "Disclosure") OR "disclosure" | |  |  |
|  | Results: Combined MeSH terms search resulted in 125 studies. | | | | | | |
| Medline | Free text | Domestic abuse | | ( (battered) W0 (women OR men) ) OR  ( (domestic OR partner OR spous*) W0 (violence OR abuse) ) | |  |  |
| Medline | Free text | Mental health setting | | ( mental health service* OR mental health setting* or psychiatric service* or psychiatric setting* ) | |  |  |
| Medline | Free text | Screening or Disclosure | | ( screen* or disclos* or detect* ) OR TI ( screen* or disclos* or detect* ) | |  |  |
|  | Results: Combined free text terms search resulted in 66 studies. Then, combined MeSH and free text terms search resulted in 177 studies and 2 potentially relevant studies. | | | | | | |

**Appendix 2**

**Table 2:** Summary of characteristics of quantitative studies

| **Author, year/ study country** | **Study design** | **Sample size and participants** | **Mental health setting** | **Data collection methodology and approach** | **Type of DA** | **Findings** |
| --- | --- | --- | --- | --- | --- | --- |
| Moodley et al., (2023)  KwaZulu- Natal, South Africa | Cross-sectional | Service users: *N*=154  Females: 100%  *M*_age_=42.7 years, 18 to 72 years of age  Black: *n*=76  White: *n*=26  Indian: *n*=32  Coloured: *n*=17  Not reported: *n*=3  Mental health condition: severe mental illness | Out-patient mental health services | -Women abuse screening tool (WAST), (Brown et al., 2000) | Verbal,  emotional, physical, sexual, and economic abuse towards females within the intimate relationships | **Prevalence of DA:**  46.6% of participants reported experiencing abuse based on the WAST scores. |
| Vranda et al. (2020)  India | Cross-sectional | Service users: *N*=100  Females: 100%  Age range: 18–55 years  Ethnicity not reported  Mental health condition: affective and non-affective psychiatric disorders | National Institute of Mental Health and Neurosciences a tertiary care psychiatric hospital (adult), outpatient and inpatient | -Danger Assessment Questionnaire  -Screening of IPV Questionnaire (Vranda & Rajaram, 2015)  -Sociodemographic and clinical profile encompassing demographic details, abuse duration, lifetime victimisation history, prior help sought, and expectations from mental health professionals | Physical, emotional, psychological, economic, and sexual abuse towards females within the intimate relationships | **Prevalence of DA:**  All women experienced moderate to severe levels of physical, emotional, economic, and sexual violence. Mean danger assessment score indicated a moderate level of danger. Half of the women experienced DA weekly, and 24% experienced it daily.  **Needs and expectations:**  90 % of women desired counselling services to address psychological distress due to violence.  40 % of women included needs for stress management, police aid, treatment for abusive partners' alcohol problems; while 22 % of women indicated needs for shelter care facilities; and 10 % highlighted a necessity for family counselling, and anger management skills for abusive partners. |
| González Cases et al. (2014)  Spain | Quantitative nonrandomized | Service users *N*=142  Females: 100%  *M*_age_=50.9 years  Ethnicity not reported  Mental health condition: Schizophrenia, personality disorder, bipolar and other psychotic disorders | Public Health outpatient mental health | -Intimate Partner Violence towards Women Questionnaire (Lasheras et al., 2008)  -Conflict Tactics Scales (CTS-1), (Straus, 1979)  -Interview: to gather comprehensive information including data on lifetime partner violence experiences, sociodemographic and clinical factors, recent psychiatric stability, and violence endured in last 6 months | Physical, Psychological, and sexual violence towards women | **Prevalence of DA:**  30.3% of the women experienced DA in the last 12 months preceding the interview.  79.6% experienced DA at some point in their lifetime. All women who experienced violence during the preceding year had also experienced previous abuse.  **Disclosure and access to resources:**  Half of the women did not disclose their situation or access any help services.  Those who sought help mainly did so with mental health services or the police.  Women who perceived themselves as abused were more likely to speak about their situation compared to those who did not perceive themselves as abused. |
| Chandra et al. (2009)  India | Quantitative nonrandomized | Service users: *N*=105  Females: 100%  *M*_age_=36.0 years  Ethnicity not reported  Mental health condition: somatoform disorder, depression, anxiety, and minor psychiatric disorders | Psychiatry outpatient  unit of National Institute of Mental Health and Neuro  Sciences | - Index of Spouse Abuse - Sexual Experiences Scale (Hudson & McIntosh, 1981) - Structured interview: women were questioned about instances of intimate partner violence, frequency of abuse, any injuries sustained, the identity and relationship of the perpetrator, and the woman's perception of the perceived reason for the abuse. | Verbal, emotional, physical, and sexual abuse towards females within intimate relationships | **Prevalence of DA:**  56% of the women reported experiencing DA and 710 % reported sexual coercion. |
| Chang et al. (2011)  USA | Quantitative nonrandomized | Service users: *N*=428  Females: *n*=270, Males: *n*=158  *M*_age_=39.7 years (males)  *M*_age_ =38.6 years (females)  Caucasian: Males: *n* = 82,  Females: *n*=140  Mental health condition: depression, PTSD, anxiety, schizophrenia, bipolar disorder | Four adult  outpatient programs, five adult inpatient units and  psychiatric emergency room | -Questions from Abuse Assessment Screen (McFarlane, 1992) to assess physical DA  -Question about sexual abuse (Currier & Briere, 2000).  -Three questions about emotional abuse adapted from the Women's Experience of Battering Scale (Smith et al., 1995).  -Provider IPV screening: asking patients if mental health professionals had inquired about physical, sexual, or emotional abuse by a partner during adulthood | Physical, Sexual and emotional abuse towards males and females within intimate relationships | **Gender differences in DA victimisation:**  Women reported significantly higher rates of DA victimisation: 63% of women reported any DA versus 32% of men.  Women were about four times as likely as men to report any DA or physical DA and 12 times as likely to report sexual DA.  **DA screening by mental health providers:**  44% of participants reported ever being asked by a mental health provider about any type of DA. While 45 % of women and 73 % of men have never been asked about DA experience. Women were twice as likely to recall being asked about all types of DA.  Participants with a history of physical DA were twice as likely to recall being asked about DA, particularly about physical DA. |
| Ruijne et al. (2021)  Netherlands | Cluster randomized controlled trial | Mental health professionals: *N*=265 in groups  Males: *M = 55.2 %*  *M*_age_=43.8 years  Ethnicity not reported | CMHT | -Searching patient files before, during, and after intervention. Detected cases were classified by type of violence and victim/perpetrator status. Referral actions were noted.  -The Better Reduction and Assessment of Violence (BRAVE) survey (Ruijne et al., 2019)  The BRAVE intervention: Response: eight hours training, workshops, and referral equipment were provided. | Sexual, physical, material  or emotional domestic violence an abuse in males and females | **Intervention outcome:**  BRAVE intervention did not lead to an increase in the detection of DA victimisation perpetration, nor did it result in an increase in DA referrals.  **Clinician readiness impact: BRAVE intervention:**  The BRAVE intervention significantly impacted clinicians' readiness to manage DA, the difference between intervention and control group was statistically significant, with an effect size of 3.21 (95% CI: 1.18-4.60), *p* < .001*. |
| Lloyd et al. (2017)  UK, Greece, Poland, Slovenia, Italy | Study 2: Mixed methods: quantitative (survey) and qualitative (focus groups) | Mental health professionals: *N*=74  Females: 90% Males: 10%  Age not reported  Ethnicity not reported | Mental health professionals (including social workers, nursers, psychologists, occupational therapists, educators, counsellors, and refugee workers) | The Physician Readiness to Measure Intimate Partner Violence Survey (PREMIS), (Short et al., 2006)-Focus group discussions on the last training session | Physical, sexual, and emotional abuse towards women within intimate relationships | **Quantitative data:** At preprogramme, 44.6% of providers agreed they could identify victims of DA, which increased to 56% at follow-up. Additionally, at preprogramme, 49.3% of providers agreed they did not have the necessary skills to discuss abuse with a victim, which decreased to 33.4% at follow up. The difference between the means at preprogramme and follow up was statistically significant (*p* <.05) for capability to identify victims of DA. |
| Burns, et al., (2022a)  South America, Europe, Japan | Cross-sectional study | Mental health professionals: *N*=321  Females: *n=*126  Males: *n*= 195  *M*_age_ = 49.78 years  Ethnicity not reported | Mental health professionals (psychiatrists and psychologists) from WHO’s Global Clinical Practice Network | - An online survey: Frequency of DA in clinical practice – by rating the frequency of using Relationship Problems and Maltreatment codes - An online survey assessed: DA-Related Training (components of training, time when the training was received and hours of training). - Knowledge and Experience of Relationship Problems: Participants rated their level of knowledge and experience regarding couples with relationship problems, including DA, on a scale of 1 to 5. | Physical or sexual DA towards women within intimate relationships | **DA-related Training:**  Less than half of participants (49.4%) received training that included all four components recommended by the WHO in DA-related training protocols.  The majority of participants (90.9%) reported receiving DA-related training both during and after their professional training, as recommended by the WHO.  **Likelihood of receiving DA-related Training:**  DA-related laws implementation in the country and frequency of using DA-related classification codes in clinical practice significantly contributed to likelihood of receiving DA training. In addition, professionals were more likely to have received more hours of DA-related training both during their professional training and after completing their professional training.  **Knowledge of DA:**  Four predictors made significant contributions to professionals’ knowledge regarding DA: Years of professional experience, DA -related laws, frequency of DA in clinical practice, and experiences of DA-related training. |
| Burns, et al., (2022b)  South America Europe Japan | Cross-sectional study | Mental Health professionals: *N*=321 (Psychiatrists *n*=173, Psychologists *n*=148)  Females: *n* =126 Males: *n* = 195  *M*_age_ = 49.78 years  Ethnicity not reported | Mental health professionals (psychiatrists and psychologists) from WHO’s Global Clinical Practice Network | - Online survey about DA-related training - ICD guidelines - Relationship Problems and Maltreatment clinical descriptions and guidelines described in vignettes and assigned to participants - Vignettes were presented to evaluate participants' diagnostic and assessment skills regarding Relationship Problems and Maltreatment and mental health and behavioural disorder in clinical scenarios - An online survey to assess DA-Related Training (Heyman et al., 2018) | Mild forms of physical and psychological abuse in heterosexual relationships | **ICD guidelines:**  There was no significant difference in accuracy of DA identification between those who use ICD-11 and those who used ICD-10 guidelines.  **Demographic factors:**  Clinicians from Japan were significantly better in identifying the absence of DA comparing to the clinicians in Europe and Latin America.  **DA-related training:**  Clinicians with DA-related training were significantly more likely to have correct responses when identifying DA compared to those without training.  Receiving DA-related training after completing professional training and within the past 5 years was associated with higher accuracy in identifying DA.  Training hours did not significantly predict the likelihood of higher number of correct responses. Clinicians who received three or four components of training performed significantly better than those without training. Receiving only one component of training or two components of training did not result in improved performance compared to no training. |
| Nyame et al., (2013)  England | Cross-sectional survey | Mental health professionals: *N*=131  Males: *n*= 69 Females: *n*=62  *M*_age_=37.5 years  White: *n*=61 | Mental Health NHS Trust, CMHT | -PREMIS | No definition was provided | **Identification:**  15% routinely asked about domestic violence. 41% identified cases in the previous 6 months.  **Response:**  27% provided information.  23% referred to counselling.  25% conducted safety assessments.  12% helped develop safety plans.  **Resource and knowledge gap:**  Over a quarter felt lacking in referral resources.  Majority lacked knowledge of support services.  **Gender differences:**  Female clinicians were more proactive in assessment and management. However, no significant gender differences were found in other PREMIS sub-scales.  **Comparison between disciplines:**  Psychiatrists showed significantly better knowledge, while nurses were significantly more ready to act. |
| Trevillion et al., (2013)  England | Mixed-methods: quantitative instruments and qualitative interviews | Service users: N=34 (intervention *n*=27, control *n*=7)  Females: *n*=33  Male: *n*=1  *M*_age_=38 years  White: *n*=13  Black and Minority ethnic origin: *n*=21  Mental health condition: depression, bipolar disorder, schizophrenia, and other psychiatric disorders  Mental health professionals: *N*=29 (intervention *n*=23, control *n*=6)  Females: *n*=17  Males (*n*=12)  *M*_age_=43 years  Ethnicity not reported | CMHT | -Composite Abuse Scale (CAS), (Hegarty et al., 1999).  -PREMIS  -LARA Intervention. Four-hour DA training for clinicians, distribution of a DA manual, six-hour mental illness training for DA advisors, direct referral pathway to DA advocacy, integrated DA advocacy provided by advisors, information campaign with posters and leaflets.   - Semi-structured interviews at follow-up to explore service users’ experiences of mental health services response to DA. | Psychological, physical, sexual, financial, or emotional between intimate partners or family members regardless of gender or sexuality | **Quantitative data:**  Clinicians in the intervention arm showed significant increase on 8 PREMIS sub/scales: perceived preparation (*p* = 0.002), perceived knowledge (*p* = 0.005), actual knowledge (*p*= 0.022), staff preparation (*p* = 0.012), legal requirement (*p* = 0.009), workplace issues (*p* = 0.009), self-efficacy (*p* = 0.004), and practice issues (*p*= 0.044).  Service users in the intervention group reported decreases in overall violence as measured by the CAS (*p* <0.001).  **Qualitative data collected from service users:**   - **Reduction in violence:**   Service users in the intervention arm credited domestic violence advisors for supporting actions to reduce harm.   - **Meeting needs:**   Intervention arm service users reported receiving assistance from advisors and clinicians for various needs, including support for children and financial aid.   - **Social inclusion:**   Intervention arm service users emphasized the intervention's role in enhancing social inclusion through engagement in community activities.   - **Process evaluation:**   Service users valued practical and emotional assistance from advisors, who actively arranged sessions and made referrals. |
| Spangaro et al., (2010)  Australia | Cross-sectional | Service users:  N=29  Females: 100%  Age: 17-60 years  Ethnicity not reported  Mental health condition: indicated as patients in table mental health condition | Mental health | - New South Wales (NSW) Health Screening Questions.  - Two surveys were conducted: one for women who tested positive for DA screening and another for those who tested negative. Both surveys asked about screening recall and the use of the provided information card. The survey for positive screens addressed service referral and utilisation post-screening, prior abuse inquiries in health services, and disclosures of recent DA experiences. The survey for negative screens aimed to uncover undisclosed abuse, termed intentional false negative responses, by asking participants their reasons for non-disclosure. | The questions in NSW mainly focus on physical and psychological abuse towards females within intimate relationships | **False responses: mental Health vs. antenatal & substance:**  Mental health patients had significantly higher odds (OR=12.2, 95% CI 3.3–46.1) of providing intentional false negative responses compared to antenatal patients. Additionally, though not statistically significant, mental health service users showed slightly elevated odds (OR=1.4) compared to drug and alcohol patients for providing false negative responses, indicating a potential trend.  **Reasons for not disclosing DA**  Underestimating the seriousness of the abuse, fear of the offender discovering, discomfort with healthcare providers, embarrassment, shame, and concerns about who else might find out. None of the respondents selected 'Thought it was own fault' as the primary reason for non-disclosure. |

**Appendix 3**

Table 3:

*Summary of characteristics of qualitative studies*

| **Author, year/ Country** | **Method** | **Sample size and participants** | **Mental health settings** | **Analytical Method** | **Study Focus** | **Type of DA** | **Key findings/results** |
| --- | --- | --- | --- | --- | --- | --- | --- |
| Vranda et al. (2018)  India | Semi-structured interviews | Service users: *N*=100  Females:  *n* =100  Age range: 18 to 56 years  Mental health condition: indicated as patients under remission without active psychopathology | Tertiary care psychiatric hospital | Frequency analysis was used to identify occurring responses and themes. | To investigate help-seeking behaviours regarding DA disclosure to mental health professionals, including reasons for disclosure or nondisclosure, post-reporting feelings, responses, and received assistance | Definition not provided but results include discussions of physical and emotional DA towards females within intimate relationships | **DA disclosure:**  62 out of 100 women were asked about DA by mental health professionals, and 10 women voluntarily disclosed DA during their visits.  **Reasons for nondisclosure:**  The most frequently mentioned reason was shame, followed by fear of not being believed, threats from partners or family, fear of re-traumatization, and disbelief that anything could change.  The least frequently reported reasons were the presence of a partner and lack of privacy, not having physical injuries, and experiencing only emotional violence.  **Reasons for voluntary disclosure:**  10 % of women voluntarily disclosed DA. Some women disclosed DA due to intolerable pain and trauma or severe ongoing violence, while others sought relief by sharing their experiences.  **Post disclosure feelings:**  Women generally felt relief or relaxation after disclosure, though some also experienced embarrassment or fear of consequences.  **Mental health professionals' response to disclosure:**  The most common response from mental health professionals was empathetic emotional support, followed by encouragement to seek help from family or friends. The least reported response was advice to attend counselling with a spouse. |
| Ruijne et al. (2020)  Netherlands. | Focus Groups | Mental health professionals: *N*=16  Females: *n*=10  Males: *n*=6  Age range: 25 – 56 years  6 out of 16 had received the BRAVE intervention | CMHT | Thematic Analysis | The study aimed to assess the acceptability, feasibility, and long-term sustainability of the BREVE intervention, alongside exploring the management and referral of domestic violence and abuse | Any occurrence of threatening actions, violence, or mistreatment (psychological, physical, sexual, financial, or emotional) among adults who are or have been involved in an intimate relationship, friendship, familial bond, or any other closely connected association (such as caregiver or roommate) | **Acceptability of the BRAVE Intervention:**  Participants who received the BRAVE intervention positively evaluated the training sessions, citing sufficient practice time, immediate applicability of skills, preference for physical attendance, and the trainer's understanding of their expertise. However, they sought more information on practical aspects like confidentiality and patient autonomy. Participants expressed a desire for more practice within the legal framework of healthcare professional-patient confidentiality, despite the training covering this aspect. They felt lacking confidence in applying their knowledge in practice. Some participants suggested a separate course specifically addressing this topic.  **Feasibility of the BRAVE Intervention:**  Participants found the BRAVE intervention feasible and utilised the learned skills and knowledge in their daily practice.  **Sustainability of the BRAVE Intervention:**  A one-off training was not sufficient to maintain effective implementation of domestic violence and abuse knowledge and skills.  **Challenges in managing and referring Victims of DA:**  CMHT professionals face moral and ethical dilemmas in managing DA, balancing patient protection with autonomy. Peer consultation or a DA consultant is desired for emotional support, though not standard practice.  **Barriers in detecting DA:**  Practical and emotional barriers hinder the detection of DA in mental health care. Procedural obstacles, such as strict intake protocols lacking DA inquiries, lead to oversight.  Emotional barriers include reluctance to address DA prematurely or in the presence of the alleged perpetrator. Safety concerns arise during home visits, often requiring colleagues for support.  The necessity of mutual trust but express fears of patient disengagement if DA is discussed. While most patients respond neutrally, some may react negatively, impacting their future care. |
| Donnelly & Holt (2020)  Ireland | Semi-structured Focus Groups | Mental health professionals: *N*=27  Males: *n*=11 Females: N=17 (please note this is the age split reported, which adds up to *N*=28, not *N*=27 which is total number of participants reported in article) | Independent acute mental health  service | Thematic analysis | To explore the experiences of Irish mental health professionals in identifying and responding to DA within a multidisciplinary team context | Not defined, but discussions of physical, emotional and sexual violence perpetrated by partner noted in results | **Barriers of identifying DA:**  Concerns about appropriateness and sensitivity, particularly during acute illness episodes.  DA detection is complicated by service users not recognizing abuse and victims intuitively gauging what can be disclosed.  Factors influencing non-identification of DA included a lack of understanding, an incidental approach, a biomedical model dominance, and lack of system support. These constructions hindered professionals' recognition and response to DA.  **Barriers of responses to DA:**  Professionals viewed it as outside the realm of mental health care, primarily a concern for social work. |
| Wilson et al. (2021)  United states | Focus Groups | Mental health professionals: *N*=23  Females: *n*= 22  Male: *n*=1  Age range: 23–58 years  Non-Hispanic White: 78%  (no specific information is provided about the remaining 22%) | CMHT | A qualitative framework approach, systematically analysing qualitative data against the Social Ecological Model (SEM) | The study aimed to understand the obstacles encountered by urban CMHT therapists who work with patients experiencing DA and Suicidal Thoughts and Behaviours, with the intention of using this information to improve and update a training program for CMHT staff | Described as intimate partner violence, no definition included. | **Barriers to addressing DA** were identified and grouped into four: individual, relational, community and societal.  **Individual barriers:**  Therapists faced personal struggles like self-doubt, fear, and burnout when dealing with patients in abusive relationships.  **Relational Barriers:**  They found it difficult to obtain accurate information from patients due to reluctance to disclose. Also, patient readiness for change was a major obstacle, with many remaining in abusive situations without considering leaving.  **Community Barriers:**  Challenges in referring patients to community resources and lack of adequate resources for DA perpetrator rehabilitation were identified.  **Societal Barriers:**  Systemic issues such as time constraints and financial barriers hindered effective counselling for DA. Also, professionals were aware of language norms and minimization tactics used by perpetrators and victims. |
| Rose et al. (2011)  England | Cross-sectional semi-structured interview study | Service users: *N*=18  Females: *n*=16  Males: *n*=2  Age range: 19–59 years  White British: *n*=9  European: *n*=1,  Black Caribbean: *n*=1  Black British: *n*=1,  Black African: *n*=1  Asian: *n*=1  Mixed Race: *n*=1  Latin American: *n*=1  Not reported: *n*=2  Mental health condition: depression, bipolar disorder, schizophrenia, borderline personality disorder, adjustment disorder, and other disorders  Mental health professionals: *N*= 20  Females: *n*=10 Males: *n*=10  Age range: 27 to 58 years | CMHT | Thematic Analysis | The aim of the study was to investigate the perspectives of both mental health service users and professionals regarding the routine inquiry about domestic violence. Additionally, the study aims to identify the factors that facilitate or hinder disclosure of domestic violence from both the perspectives of mental health service users and professionals. | Domestic violence was defined as abuse by another person either  psychologically, physically, sexually, financially, or emotionally,  within their home or relationships | **Service users' barriers to disclosure of domestic violence:**  Fear of disclosure due to disbelief, family disruption, and immigration status consequences.  Blaming attitudes, self-blame, and shame hindered disclosure.  Psychological distress closely linked to experiences of domestic violence.  Some users did not recognise abuse until later stages.  Perpetrators hindered disclosure through isolation and manipulation.  Services’ failure to respond to abuse signs.  **Service users’ suggested factors that can facilitate disclosure:**  The importance of engagement between professionals and service users.  **Mental health professionals' barriers to enquiry of domestic violence:**  Barriers to asking about abuse: role uncertainty, time constraints, fear of offense.  Mixed confidence levels in addressing the issue.  Focus on mental health issues sometimes overshadowed domestic violence discussions.  Challenges with gender mismatches and cultural norms.  **Mental health professionals’ suggested factors that can facilitate enquiry:**  Therapeutic engagement aided discussions on DA. |
| Gillespie et al., (2022)  Australia | Descriptive design, semi-structured  interviews | Mental health professionals: *N*=12 | Range of teams across mental health department of a public hospital and health service | Grounded theory approach and reflexive thematic analysis | The aim was to investigate the experiences and viewpoints of mental health professionals regarding screening for DA, as well as to recognize the obstacles and facilitators in the processes of screening, identification, response, and providing supportive intervention | Described as actions carried out by a partner or family member resulting in physical, sexual, or psychological harm, domestic violence encompasses behaviours such as aggression, coercion, and control | **Barriers to screening for DA included:**  Insufficient training availability of education on DA for professionals.  A lack for understanding of the basics in DA screening and safety.  DA screening is not given priority in their daily practice.  Mental health needs of patients are prioritized over inquiring about DA.  Attitudes towards DA are influenced by a lack of education about it. |
| England | Individual in depth  interviews | **Service users:** *N*=24  Females: *n*=18  Males: *n*=6  Age range: 19 – 59 years  White British: *n*=12  European: *n*=1  Black Caribbean: *n*=5  Black British: *n*=1  Black African: *n*=2  Asian: *n* = 1  Mixed Race: *n*=1  Latin American: *n*=1  Mental health condition: bipolar disorder, depression, borderline personality disorder, schizophrenia, adjustment disorder, and other disorders  **Mental health professionals:** *N*=25  Females: *n*=15 Males: *n*=10  Age range: 27-58 years | CMHT or voluntary sector services | Thematic Analysis | The study aimed to explore attitudes toward routine inquiry, current identification, and response methods to domestic violence within mental health services, and strategies for enhancing responses to abuse | Same definition as in the study by Rose et al. (2011) | **Service users’ acceptability of routine enquiry:**  Service users who experienced and who did not experienced DA found routine inquiry acceptable, stating it would facilitate disclosure, raise professional awareness of abuse, aid in identifying factors contributing to mental health symptoms, and increase awareness of violence risks among individuals with severe mental illness.  **Service users’ experience of response to domestic violence disclosure:**  Some received encouragement to discuss their experiences, leading to a sense of relief and a desire for further conversation. However, many reported that professionals were aware of the abuse but did not acknowledge it or were unreceptive to disclosures. Service users had difficulties in accessing support services, with some anticipating or experiencing discrimination due to mental health issues. Some received ongoing assistance from professionals, but several others failed to receive the support.  **Mental health professionals’ acceptability of routine enquiry:**  Most mental health professionals found routine enquiry into DA acceptable and integrated it into their clinical assessments as part of their duty of care. However, some lacked confidence addressing it, feeling untrained in knowing what questions to ask or how to handle the information. A minority questioned its relevance to their role.  **Mental health professionals’ views on responding to domestic violence:**  Professionals discussed the necessity of integrated discussions on domestic violence into routine practice and being sensitive and available to service users when it comes to identifying DA. Professionals' experiences in reporting and making referrals, with mixed views on procedures and challenges in maintaining confidentiality were presented. Both service users and professionals emphasized the need for improved collaboration among agencies and specialized services tailored to individuals experiencing violence and mental illness. |
| Spangaro et al. (2011) Australia | Focus Group | Mental health professionals: *N*=7 | Information not provided | Inductive approach | The study aimed to understand challenges and enablers of New South Wales IPV Screening Questions | The questions of New South Wales Health Screening are mainly focused on physical and psychological violence | **Challenges in DA screening:**  Establishing patient privacy.  Inconsistent child protection responses cause irregular reporting.  Frustration expressed by professionals regarding women who chose to remain in abusive relationship.  **Enablers for DA screening:**  Efficient screening questions that do not consume much of the clinician’s time.  Utilising a form with questions to facilitate the recording responses.  Ability to visually demonstrate the questions to patients.  **Impact of DA screening on practice**:  Professionals have acknowledged the relief experienced by patients when they can openly discuss DA and gain a better understanding of their mental health symptoms.  DA screening has broadened the scope for professionals to ask sensitive questions of their patients. |
